# Electrochemical detection of uric acid in undiluted human saliva using uricase paper integrated electrodes

**DOI:** 10.1038/s41598-022-16176-5

**Published:** 2022-07-14

**Authors:** Seong Hyun Han, You-Jung Ha, Eun Ha Kang, Kichul Shin, Yun Jong Lee, Gi-Ja Lee

**Affiliations:** 1 Department of Medical Engineering, Kyung Hee University, Graduate School, Seoul, 02447 Republic of Korea; 2 Division of Rheumatology, Department of Internal Medicine, Seoul National University Bundang Hospital, Seongnam-si, Gyeonggi-do 13620 Republic of Korea; 3 Division of Rheumatology, Seoul Metropolitan Government-Seoul National University Boramae Medical Centre, Seoul, 07061 Republic of Korea; 4 Department of Medical Device Development, Seoul National University Graduate School, Seongnam-si, Gyeonggi-do 13605 Republic of Korea; 5 Department of Biomedical Engineering, College of Medicine, Kyung Hee University, #1 Kyungheedae-ro, Dongdaemun-gu, Seoul, 02447 Republic of Korea

**Keywords:** Sensors, Diagnostic markers, Sensors and probes

## Abstract

In this study, we introduce a uricase-immobilized paper (UOx­-paper) integrated electrochemical sensor for detection of uric acid (UA) in saliva. The UOx was immobilized on the detection zone in the wax-patterned paper substrate. This UOx-paper was integrated with a Prussian blue­-modified, screen-printed carbon electrode after electropolymerization of o-phenylenediamine to construct an electrochemical cell for small-volume (20 μL) of samples. First, we optimized the fabrication conditions of UOx-paper. Next, the amperometric response of the UOx-paper-based electrochemical UA sensor was analyzed using a known concentration of UA standard solution in artificial saliva at an applied potential of − 0.1 V (versus Ag pseudo-reference electrode). The UOx-­paper based electrochemical UA sensor showed a sensitivity of 4.9 μA·mM^−1^ in a linear range of 50 to 1000 μM (R^2^ = 0.998), high selectivity and good reproducibility, as well as a limit of detection of 18.7 μM (0.31 mg/dL) UA. Finally, we quantified the UA levels in undiluted saliva samples of healthy controls (n = 20) and gout patients (n = 8). The levels were correlated with those measured with conventional salivary UA enzymatic assays as well as serum UA levels. The UOx-paper-based electrochemical UA sensor is a user-friendly and convenient tool to assess salivary UA levels.

## Introduction

Uric acid (UA), the final product of purine metabolism in the human body, plays an important role in a variety of physiologic and pathologic conditions, including gout^1–3^. UA is an antioxidant and hypouricemia has been reported to be associated with immune-mediated or degenerative neurological diseases such as multiple sclerosis, Parkinson’s disease, or Alzheimer’s disease^3^. Recurrent aphthous stomatitis and oral lichen planus were also associated with low levels of salivary UA^4,5^. However, hyperuricemia contributes to the development and progression of gout, metabolic syndrome, chronic kidney disease or cardiovascular diseases^3^. Although hyperuricemia does not always induce gout and the diagnosis of gout is not based on hyperuricemia alone, gout develops in subjects with hyperuricemia leading to the deposition of monosodium urate crystals in tissues. Recent guidelines for the management of gout recommend that urate-lowering therapy should be optimized to achieve and maintain a serum UA level < 5–6 mg/dL, but not < 3 mg/dL^6,7^. Therefore, monitoring of serum UA is indispensable for the diagnosis, treatment, and follow-up of hyperuricemia and gout. However, venupunture is invasive and can cause complications including injuries and bleeding. Further, analysis of serum UA requires a laboratory setting using specialized equipment.

Salivary diagnostics have attracted increased attention in the fields that utilize point-of-care testing (POCT) and in clinical applications for monitoring diseases frequently and easily, in addition to predicting post-treatment outcomes because saliva reflects the physiological and pathological status of the body^8,9^. UA is mainly produced in the liver and intestines. Most UA is eliminated via kidneys and intestines through urate transporters. However, organic anion and urate transporters are also expressed in the salivary glands^10^. Additionally, several clinical studies reported a linear relationship between serum and salivary UA levels in most cases, suggesting the potential of salivary UA determination as an alternative for blood tests^1,2,11,12^. Various analytical methods including high-performance liquid chromatography (HPLC)^13^, capillary electrophoresis (CE)^14^, and enzymatic colorimetric assay kits^15–18^ have been developed for the detection of salivary UA. However, they are not suitable for daily personal use because they are usually time-consuming and requiring expensive instruments and experts. Although some commercially available enzymatic assay kits can be used for various types of biological matrices such saliva, serum, and urine^2^, they can be influenced by other interferences such as vitamin C, lipid, or endogenous peroxidase that are present in biological samples, and eventually cause false results^2,19–22^. Besides, they have a short use-by date because peroxidase within reagents is incompatible with preservatives such as sodium azide^23^. As a result, an overdue reagent may result in a decrease of their sensitivity^2,24^.

Electrochemical sensors received significant attention due to practical advantages, including high sensitivity, rapid response time, portability, low cost, and ease of operation for detecting salivary UA^25–27^. Kim et al. reported a wearable salivary UA mouthguard biosensor using uricase-modified screen-printed electrode (SPE) system with integrated wireless electronics^28^. Although this mouthguard biosensor might be able to monitor salivary UA level in real-time continuously, its use is limited due to discomfort and biocompatibility issue of electronics. Huang et al. reported a paper-based electroanalytical device for salivary UA analysis^29^. They fabricated poly (3,4-ethylene dioxythiophene)–graphene oxide composites on indium tin oxide (ITO) substrates as the working electrodes and covered the prepared electrode with a piece of paper to construct a thin-layer electrochemical cell. Although this device showed high sensitivity, it is inconvenient to use because it requires separate reference and counter electrodes. Therefore, an easy, rapid, cost-effective and sensitive method is needed for the analysis of salivary UA for POCT of UA-associated diseases.

**Figure 1:**
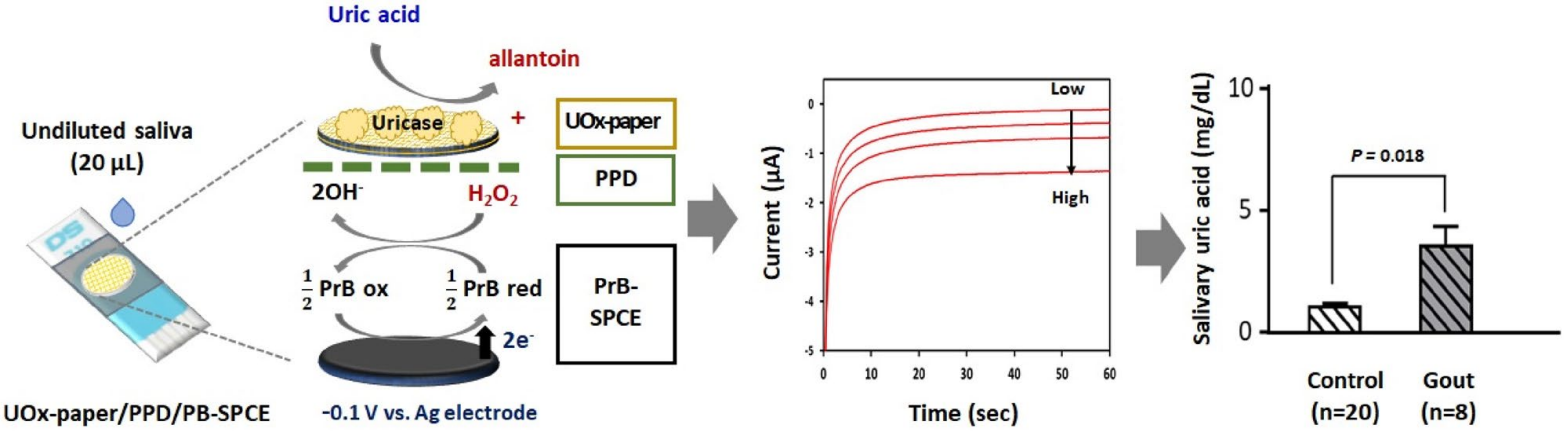
Schematic representation of the sensing principle and resulting current signal of the UOx-paper-based electrochemical UA sensor, and uric acid concentrations (mg/dL) in human saliva samples.

In this study, we fabricated a facile and effective electrochemical UA sensor based on a uricase (UOx)-immobilized paper (UOx-paper) and a Prussian blue (PrB)-modified, screen-printed carbon electrode (PrB-SPCE). To improve the selectivity and anti-biofouling of the electrode, o-phenylenediamine (o-PD) was electropolymerized on the PrB-SPCE (PPD/PrB-SPCE). The UOx-paper using a wax-patterned lens cleaning tissue with a single circle was integrated with the PPD/PrB-SPCE to construct an electrochemical cell for small-volume samples. First, we optimized the fabrication conditions for UOx immobilization on the paper. Next, we evaluated the analytical performance of the UOx-paper integrated electrochemical UA sensor, based on sensitivity, selectivity, reproducibility, and stability, in artificial saliva. Finally, the UOx-paper integrated electrochemical UA sensor was evaluated by determining the concentration of UA in undiluted saliva samples obtained from non-gout controls (n = 20) and patients with gout (n = 8), comparing serum UA levels and those measured with conventional Salimetrics® salivary UA enzymatic assay kits. The UOx-paper integrated electrochemical sensor for detection of salivary UA is schematically represented in Fig. 1.

## Materials and methods

### Materials and chemicals

UA (≥ 99.0%), UOx (4 units/mg of *Candida sp*.), o-PD, sodium sulfate (Na_2_SO_4_), sodium chloride (NaCl), calcium chloride (CaCl_2_), potassium chloride (KCl), citric acid, potassium thiocyanate (KSCN), ammonium chloride (NH_4_Cl), potassium phosphate monobasic (KH_2_PO_4_), potassium phosphate dibasic (K_2_HPO_4_), L-lactic acid (LA), D-glucose (Glu), L-ascorbic acid (AA), acetaminophen (AP), bovine serum albumin (BSA) and glutaraldehyde (GA) solution were purchased from Sigma Aldrich (St. Louis, MO, USA). Whatman lens cleaning tissue (grade 105) was ordered from GE Healthcare Life Sciences (Pittsburgh, PA, USA). All reagents were of analytical grade and used without further purification. All aqueous solutions were freshly prepared using deionized water (DW) with 18.2 MΩ· cm resistivity.

### Instrumentation

The wax-patterned paper was prepared using a XeroxColorQube 8570 N printer (Fuji Xerox, Tokyo, Japan) with AutoCAD software (Autodesk, San Rafael, CA, USA) for pattern design and a BF-150C drying oven (DAIHAN Scientific, Seoul, Korea) for wax impregnation. All electrochemical experiments, including cyclic voltammetry and chronoamperometry (CA), were carried out with a Compactstat (Ivium Technology, Eindhoven, The Netherlands) at room temperature (RT). PrB-SPCE containing a PrB-modified carbon working electrode (4 mm in diameter), a carbon counter electrode and an Ag pseudo-reference electrode was purchased from DropSens (DRP-710, Llanera, Asturias, Spain).

### Preparation of UOx-immobilized paper

The lens cleaning tissue with hydrophobic wax barrier measured 11 mm in width and 15 mm in length for one electrode. The hydrophilic zone was 8 mm in diameter (Fig. S1[A] in Supplementary Information (SI)). Uniform impregnation of wax on the paper was performed in a BF-150C drying oven at 80 °C for 120 s. Finally, the wax patterned paper was removed from the oven, and cooled rapidly to RT.

To immobilize UOx within the lens cleaning tissue, 30 units of UOx (37.5 mg/mL) were mixed with 2 mg of BSA and 1 μL of 8% glutaraldehyde solution in 200 μL of potassium phosphate buffer (PB, 0.1 M, pH 7.0), as shown in Fig. S1(B) in SI. Next, 10 μL of mixed solution was dropped on the wax patterned paper and dried in a clean room (23.5 ± 1.0 °C, 25.0 ± 5.0%) for 40 min. To eliminate the unbound enzyme, this paper was washed with 0.1 M PB. Finally, the UOx-paper was dried in a clean room (23.5 ± 1.0 °C, 25.0 ± 5.0%) for 30 min.

### Fabrication of electrochemical UA sensor and electrochemical measurements

Prior to fabrication of the UA sensor, we confirmed the electrochemical properties of PrB-SPCE via cyclic voltammetry in KCl solution with a potential range from − 0.1 to 0.4 V (vs. Ag pseudo-reference electrode) and a scan rate of 50 mV/s. Next, poly(o-PD) (PPD) was deposited on the PrB-SPCE by polymerization of o-PD at 0.7 V (vs. Ag pseudo-reference electrode) for 100 s in 0.1 M PB (pH 7.0) solution containing 10 mM o-PD and 5 mM sodium sulfate to minimize biofouling and interference from saliva constituents^28^. Next, a section of double-sided adhesive tape with a punched hole (8 mm in diameter) was attached to the electrode. Finally, the tape was covered with a UOx-immobilized paper to store the sample solution as well as electrically connect the three-electrode system for electrochemical detection.

The CA technique was used to evaluate the UA detection sensitivity of the UOx-paper/PPD/PrB-SPCE. A standard solution of UA was prepared in artificial saliva consisting of 5 mM NaCl, 1 mM CaCl_2_, 15 mM KCl, 1 mM citric acid, 1.1 mM KSCN, and 4 mM NH_4_Cl in DW^28^. The pH of artificial saliva was adjusted to 6.7. Various concentrations of UA were detected using the CA technique at an applied potential of − 0.1 V (vs. Ag pseudo-reference electrode) for 60 s after 1 min of incubation in the standard solution. The calibration curve was obtained from CA measurements in 20 μL of UA at a concentration range of 50 to 1000 μM in artificial saliva.

### Study participants and sample collection

This study and sample collection were approved by the Ethics Committee of Seoul National University Bundang Hospital (IRB No. B-1911-577-303) and written informed consent was obtained from all participants. We enrolled 8 male patients with gout (age (mean ± standard error of mean (SEM)), 39.1 ± 3.0 years) and 20 non-gout males (age 40.8 ± 6.4 years). Serum and saliva samples were simultaneously collected under fasting conditions. Additionally, we obtained 16 paired-samples from patients with gout before and after starting urate-lowering therapy (febuxostat (n = 4), allopurinol (n = 3), or benzbromarone (n = 1)). The duration of treatment was 2.9 ± 1.1 months. Unstimulated whole saliva samples were collected by passive drooling into a plastic tube. Venous blood samples were centrifuged (1500×*g* for 15 min at RT) to obtain serum. The whole saliva samples were centrifuged at 4500×*g* for 10 min at 4 °C. All samples were frozen at − 80 °C until analysis. Serum UA levels were determined using commercially available enzymatic colorimetric assay kits (Beckman Coulter Inc., Brea, C.A, USA) according to the manufacturer's instruction. For salivary UA analysis, 20 μL of undiluted saliva sample was first dropped on the UOx-paper/PPD/PrB-SPCE. After incubation for 60 s, CA measurement was performed at − 0.1 V (vs. Ag pseudo-reference electrode) for 60 s. The UA concentration in saliva sample was calculated from the slope of the calibration curve. Additionally, salivary UA levels were measured using a salivary UA enzymatic assay kit (Salimetrics LLC, Philadelphia, PA, USA) according to the manufacturer's instruction.

### Statistical analysis

Continuous variables are presented as mean ± SEM. Independent sample t-test was used for comparison of the two groups. Paired t-test was used to compare UA levels before and after urate lowering therapy. One-way ANOVA was used to compare UA levels across 3 groups and Fisher’s exact test was performed to analyze categorical data. Pearson’s correlation coefficients were calculated to analyze the relationship between serum and salivary UA levels or between salivary UA levels measured by the prepared UA sensor and conventional method. A *p* value of 0.05 was considered for statistical significance. Statistical analysis was performed using the IBM® SPSS Statistics, Version 25 (IBM Corporation, Armonk, NY, USA).

## Results and discussion

### Electrochemical characterization of PPD/PrB-SPCE toward H_2_O_2_ in artificial saliva

**Figure 2:**
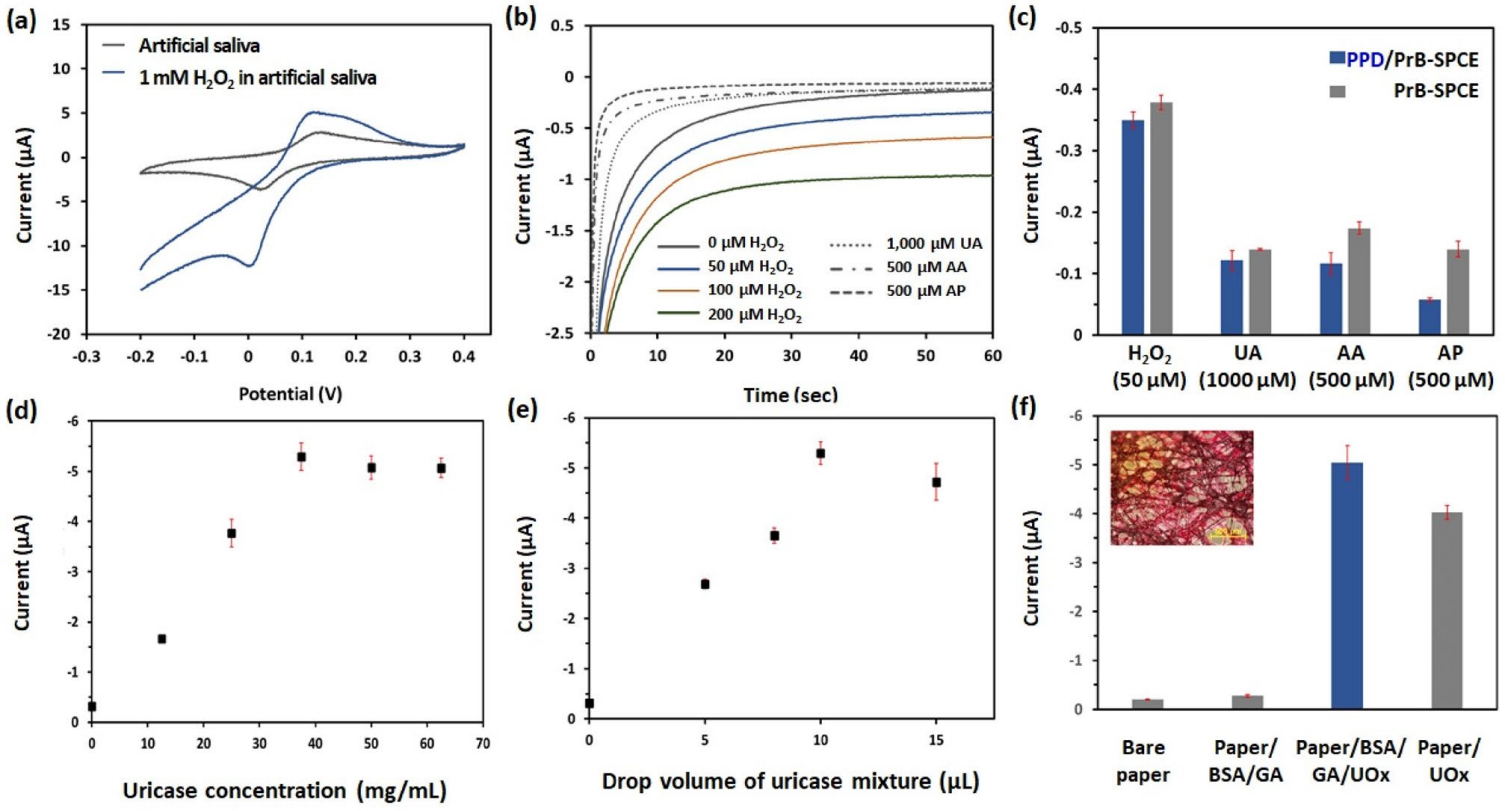
(**a**) Cyclic voltammograms of PPD/PrB-SPCE in an artificial saliva solution with and without 1.0 mM H_2_O_2_ at a potential ranging from − 0.2 to 0.4 V (vs. Ag pseudo-reference electrode) and a scan rate of 50 mV/sec. (**b**) Current response to 0, 50, 100, and 200 μM H_2_O_2_ of PPD/PrB-SPCE compared with response to common electroactive interferences including 1000 μM UA, 500 μM AA, and 500 μM AP. (**c**) Comparison of the electrochemical response of PPD/PrB-SPCE and PrB-SPCE to 50 μM H_2_O_2_ and 1000 μM UA, and 500 μM AA, and 500 μM AP, respectively, at − 0.1 V (vs. Ag pseudo-reference electrode). Effect of (**d**) UOx concentration, (**e**) drop volume of UOx mixture, and (**f**) UOx immobilization method on the analysis of 1000 μM UA. Error bars represent standard deviation of the mean (n = 3).

Typically, UA biosensors have utilized enzymes such as UOx with high specificity for UA. However, as UOx-based amperometric detection of UA generally requires a relatively high potential (> + 0.65 V) to measure the hydrogen peroxide (H_2_O_2_) product, it is subject to various electroactive interferences. PrB or ferric hexacyanoferrate has been referred to as an “artificial peroxidase”, because it can enhance electron transport and catalyze the reduction of H_2_O_2_ at low overpotential^30,31^. Therefore, PrB-SPCE provides selective cathodic detection of H_2_O_2_ produced by the enzymatic reaction of UA. However, the PrB may decompose in neutral or weakly alkaline solutions^32^. Besides, saliva is a complex and difficult matrix to manage due to its high viscosity and protein assembly, as well as other electroactive species^33^. To improve the stability, selectivity and biocompatibility of PrB-SPCE, we introduced an external protective polymer membrane such as PPD on PrB-SPCE via electropolymerization. PPD membrane is known for its ability to penetrate low-molecular-weight compounds such as H_2_O_2_ and reject other electroactive species such as AA and AP, as well as prevent biofouling on the electrode^28,34^. To characterize the electrocatalytic property of PPD/ PrB-SPCE toward H_2_O_2_, we performed cyclic voltammetry measurements in artificial saliva with and without 1 mM H_2_O_2_ in the potential range of − 0.20 to + 0.40 V (vs. Ag pseudo-reference electrode) at a scan rate of 50 mV/sec. As shown in Fig. 2a, the PPD/PrB-SPCE exhibited the characteristic Prussian White (PrW)/PrB redox activity (0.02/0.13 V) in the artificial saliva solution. The cathodic peak current increased to − 12.3 μA at − 0.00 V in 1 mM H_2_O_2_ solution. To confirm the electrochemical performance of PPD/PrB-SPCE toward H_2_O_2_, the CA technique was used at an applied potential of − 0.1 V (vs. Ag pseudo-reference electrode). The selectivity of PPD/PrB-SPCE was confirmed by the current response of H_2_O_2_ in the presence of physiological levels of the relevant electroactive constituents of saliva including UA, AA, and AP. As shown in Fig. 2b and 2c, the interference currents due to UA (1000 μM), AA (500 μM), and AP (500 μM) were negligible, compared with the strong response due to H_2_O_2_ (50, 100, and 200 μM). In particular, the current responses of AA (− 0.12 ± 0.02 μA, [mean ± standard deviation]) and AP (− 0.06 ± 0.002 μA) on PPD/PrB-SPCE were significantly lower than those on PrB-SPCE (− 0.17 ± 0.01 μA for AA and − 0.14 ± 0.01 μA for AP, respectively). However, the cathodic current of H_2_O_2_ (− 0.35 μA ± 0.01 μA) on PPD/PrB-SPCE and PrB-SPCE (− 0.38 ± 0.01 μA) was similar. This result indicates that PPD/PrB-SPCE has high selectivity for the detection of H_2_O_2_ without any interference effect by possible electroactive species in saliva. In addition, the external PPD membrane did not inhibit the permeability of H_2_O_2_ toward PrB-SPCE.

### Fabrication and optimization of UOx-paper

Paper-based analytical platforms have several advantages including low cost, simple fabrication, and are easy to use in many applications such as biochemical and analytical sensors^35^. In addition, paper can be easily designed according to the desired pattern by wax printing with a non-toxic reagent. To use paper as a bioanalytical tool for UA detection, we designed a single zone that was integrated to the three-electrode system of SPCE. Next, we immobilized UOx within the hydrophilic zone of wax-patterned lens paper for use as a sample absorption area as well as electrochemical cell to facilitate the reaction between UOx and UA within samples. Therefore, it was necessary to optimize the experimental conditions, including paper type, UOx concentration, and volume of UOx mixture for efficient immobilization of UOx on the paper.

First, we optimized the types of paper including Whatman No. 1, No. 114, and lens cleaning tissues No. 105 for immobilization of UOx. As the pore size and thickness differ with the type of paper (Table S1), the result of detection varies due to the differences in solution flow rate on the paper surface and material uniformity^36^. Therefore, appropriate paper selection appears to be critical to improve the current signal of paper-based biosensors. The effect of paper type was investigated according to the cathodic current response of UOx-paper/PPD/PrB-SPCE on 1000 μM UA at − 0.1 V (vs. Ag pseudo-reference electrode). As shown in Fig. S1(C), the UA current was the highest in lens cleaning tissue, which was the thinnest and carried the widest pores. To enhance the electrochemical response of the UOx-paper/PPD/PrB-SPCE, we optimized the UOx concentration and the drop volume of UOx mixture. Figure 2d shows the change in the cathodic current response of UOx-paper/PPD/PrB-SPCE at − 0.1 V (vs. Ag pseudo-reference electrode) according to the concentration of UOx (0, 12.5, 25, 37.5, 50, and 62.5 mg/mL). The UA current increased with increase in the UOx concentration from 12.5 to 37.5 mg/mL. As the concentration of UOx increased further, the cathodic current of the UOx/PPD/PrB-SPCE decreased. In addition, when the UOx concentration was fixed at 37.5 mg/mL, the optimum drop volume of UOx mixture was found to be 10 μL (Fig. 2e)). The increased drop volume of UOx mixture resulted in decreased current signal.

GA is one of the most widely used bifunctional reagent for intermolecular cross-linking of protein which forms covalent bonds from the reaction between aldehydes of the cross-linker and amines of the protein^37,38^. But when enzymes are directly cross-linked to GA, they tend to lose activity. Therefore, mild cross-linking with GA using a feeder rich in amino groups such as BSA can increase the stability of enzyme by reducing the chemical modification of internal amino groups of the enzyme^39^. Besides, it can reduce the porosity of the film and, thus, increase the responsiveness of the film^40^. As a result, the UOx-paper prepared using GA and BSA as a crosslinker and stabilizer generated a higher current (− 5.04 ± 0.35 μA) than the paper prepared using only UOx (− 4.03 ± 0.14 μA) (Fig. 2f). Inset shows the stained image of UOx-paper using Ponceau S, which is generally used for the detection of protein on cellulose acetate and nitrocellulose membranes^41^. The proteins on the UOx-paper stained red with Ponceau S dye. Based on the current signal and stained image of UOx-paper, we confirmed that UOx was successfully immobilized on the paper. The optimum conditions of 37.5 mg/mL UOx and 10 μL of UOx mixture with BSA and GA on the lens cleaning tissues No. 105 were used in further experiments.

### Analytical performance of UOx-paper/PPD/PrB-SPCE towards UA

**Figure 3:**
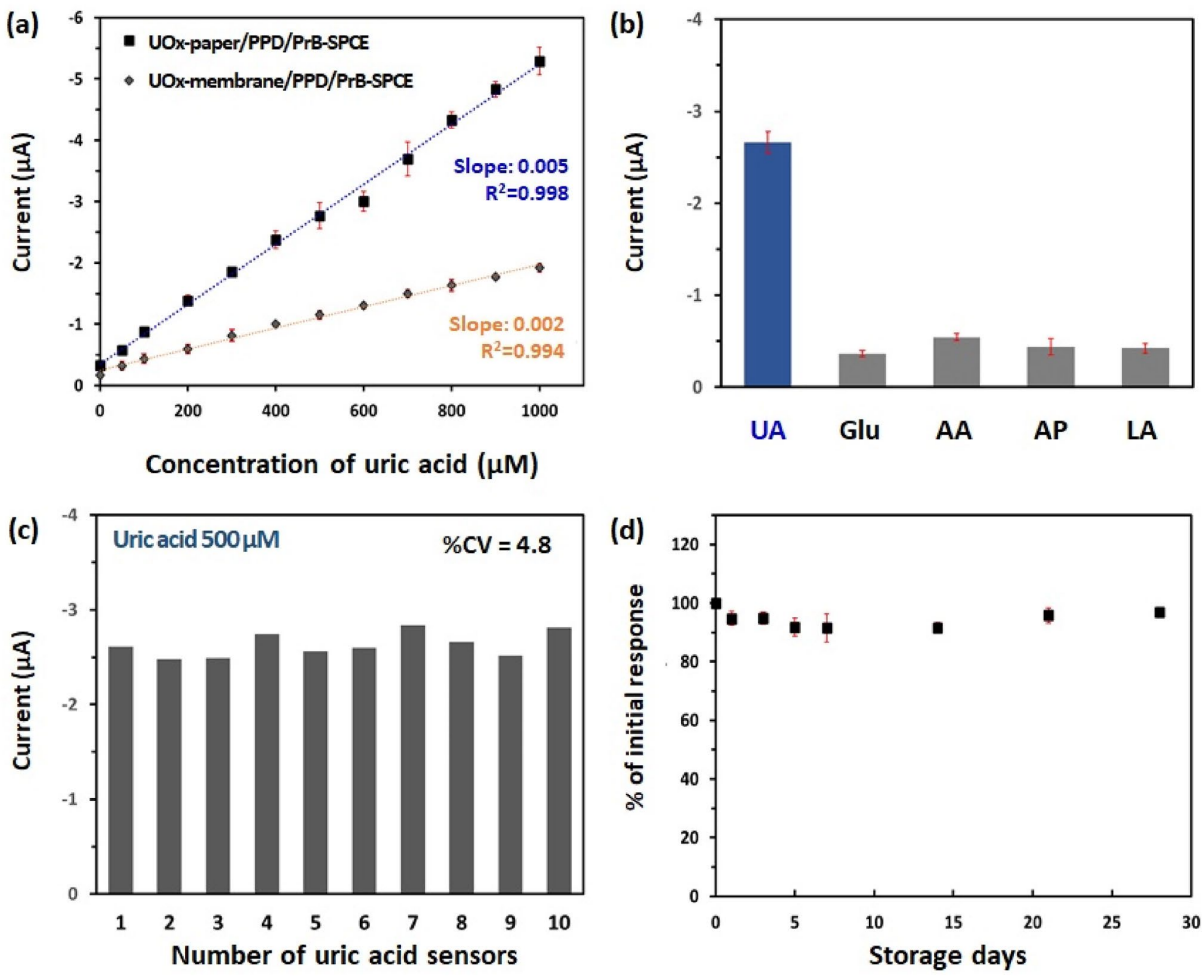
(**a**) The calibration curve of the cathodic current vs. UA concentration in the comparison of UOx-paper/PPD/PrB-SPCE and UOx-membrane/PPD/PrB-SPCE at − 0.1 V (vs. Ag pseudo-reference electrode), respectively. (**b**) The current response to 500 μM UA of UOx-paper/PPD/PrB-SPCE at − 0.1 V (vs. Ag pseudo-reference electrode) compared with response to common electroactive physiological interferences including 800 μM glucose, 200 μM AA, 100 μM AP and 1000 μM LA. (**c**) Reproducibility of UOx-paper/PPD/PrB-SPCE with different fabrication dates, based on current response to 500 μM UA in artificial saliva at − 0.1 V (vs. Ag pseudo-reference electrode). (**d**) Stability of UOx-paper/PPD/PrB-SPCE based on the current response to 500 μM UA stored for 28 days at 4 °C. Error bars represent standard deviation of the mean (n = 3).

We investigated the analytical performance of UOx-paper/PPD/PrB-SPCE for UA detection using the CA technique. The current responses of UOx-paper/PPD/PrB-SPCE in 20 μL of artificial saliva solutions containing various concentrations of UA (0­1000 μM) were measured at an applied potential of − 0.1 V (vs. Ag pseudo-reference electrode). As shown in Fig. 3a, the cathodic current of UOx-paper/PPD/PrB-SPCE increased with increasing UA concentration. The response of the UOx-paper/PPD/PrB-SPCE was linear with respect to UA concentration up to 1000 μM (R^2^ = 0.998), with a detection limit of 13.3 μM according to the standard deviation of the blank and the slope method (3 s_bl_/slope)^42^ and a detection sensitivity of 5.0 μA·mM^−1^ (39.8 μA·mM^−1^·cm^−2^). However, the current response of UOx-membrane/PPD/PrB-SPCE, in which UOx was directly immobilized on PPD/PrB-SPCE using GA and BSA, was smaller than that of UOx-paper/PPD/PrB-SPCE. As a result, the slope of the calibration curve of the UOx-paper/PPD/PrB-SPCE was about threefold higher than that of UOx-membrane/PPD/PrB-SPCE (1.7 μA·mM^−1^). Table 1 presents a comparison of the sensing performance of our UOx-paper/PPD/PrB-SPCE with that of other UOx-based SPCEs for detection of salivary UA. The performance of our UA sensor was good in terms of low sample volume and wide linear range, together with moderate sensitivity. In particular, its detection range included all concentrations of salivary UA from healthy control to gout patients.

**Table 1:** Comparison of analytical performance of UOx-paper/PPD/PrB-SPCE with other UOx-treated electrodes.

Electrode	Tested sample	Sample volume (μL)	Operating potential (V)	Linear range (μM)	LOD (μM)	Sensitivity (μA·cm^−2^·mM^−1^)	References
UOx/MWCNTs/SPE	Saliva	100	0.4	5–1000	0.33	122.2	^46^
UOx/Os-HPR redox polymer/SPE	Saliva	60	0	10–400	–	170 (nA mM^−1^)	^27^
UOx/PVA-SbQ Polymer/SPCE	Saliva	–	0.33	12–100	–	155	^47^
UOx/PPD/PrB/SPE	Saliva	100	− 0.3	50–1000	–	25.7	^2,8^
UOx-paper/PPD/PrB-SPCE	Saliva	20	− 0.1	50–1000	17	37.5	This work

*MWCNTs* multi-walled carbon nanotubes, *Os-HRP* osmium-wired horseradish peroxidase, *PVA-SbQ* pyridinium methosulfate acetal, *LOD* limit of detection.

To evaluate the selectivity of the UOx-paper/PPD/PrB-SPCE, we compared the current responses of 500 μM UA with that of other potential interfering substances, including 800 μM Glu, 200 μM AA, 100 μM AP and 1000 μM LA. As shown in Fig. 3b, only UA induced a dramatic change in the electrical current, while the interference currents caused by Glu, AA, AP, and LA were negligible. This result showed that UOx-paper/PPD/PrB-SPCE had high selectivity for the detection of UA without effects associated with possible interferents. In addition to interferents, nonspecific biomolecule or microbial adsorption is a persistent and pervasive threat for interfaces exposed to biological fluids, subsequently may be either partially or completely impaired the function of biosensors^43^. PPD is known for having anti-biofouling property in proteinaceous media, possibly due to its greater compactness^44,45^. We tested an anti-biofouling ability of UOx-paper/PPD/PrB-SPCE using artificial saliva and real saliva samples. As shown in Fig. S2, the current response of UOx-paper/PPD/PrB-SPCE to UA (300 μM) which was spiked in real saliva retained to 86% and 92% of that in artificial saliva. But UOx-paper/PrB-SPCE without PPD showed 63% and 68% of its current response in real saliva relative to artificial saliva. Therefore, we thought that PPD might be effective to minimize nonspecific adhesion of protein on the electrode surface.

In addition, we investigated the reproducibility of UOx-paper/PPD/PrB-SPCE by measuring the current response to UA (500 μM) using 10 sensors on different fabrication dates. As the coefficient of variation (CV) of our UA sensor was 4.8% (Fig. 3c), the UOx-paper/PPD/PrB-SPCE was highly reproducible. The stability was tested by evaluating the current response of UOx-paper/PPD/PrB-SPCE to 500 μM UA during 28 days of storage at 4 °C. As shown in Fig. 3d, there was no significant change in current in 28 days (~ 97% of the initial response), suggesting the stability of the UOx-paper/PPD/PrB-SPCE.

### Salivary UA levels in patients with gout compared with non-gout controls

Saliva is a biological fluid that is used in clinical diagnosis and management of patients, because of its many advantages, including non-invasive collection, easy-to-use sample, and inexpensive storage^48^. However, its routine use is limited by further dilution of analytes, interference by food, or periodontal or salivary gland health status. In particular, saliva is a difficult and complex matrix for biological analysis because many components in saliva can affect the response of the analyte of interest^49^. Therefore, a highly sensitive and selective method is required for detection of UA in saliva.

To evaluate the feasibility of our UA sensor for POCT, we measured salivary UA concentrations of non-gout controls (n = 20) and patients with gout (n = 8). In addition, in case of patients with gout, we determined salivary UA levels before and after urate-lowering therapy. The results were compared with those of commercial salivary UA enzymatic assays (Salimetrics®), together with serum UA levels as reference values.

**Figure 4:**
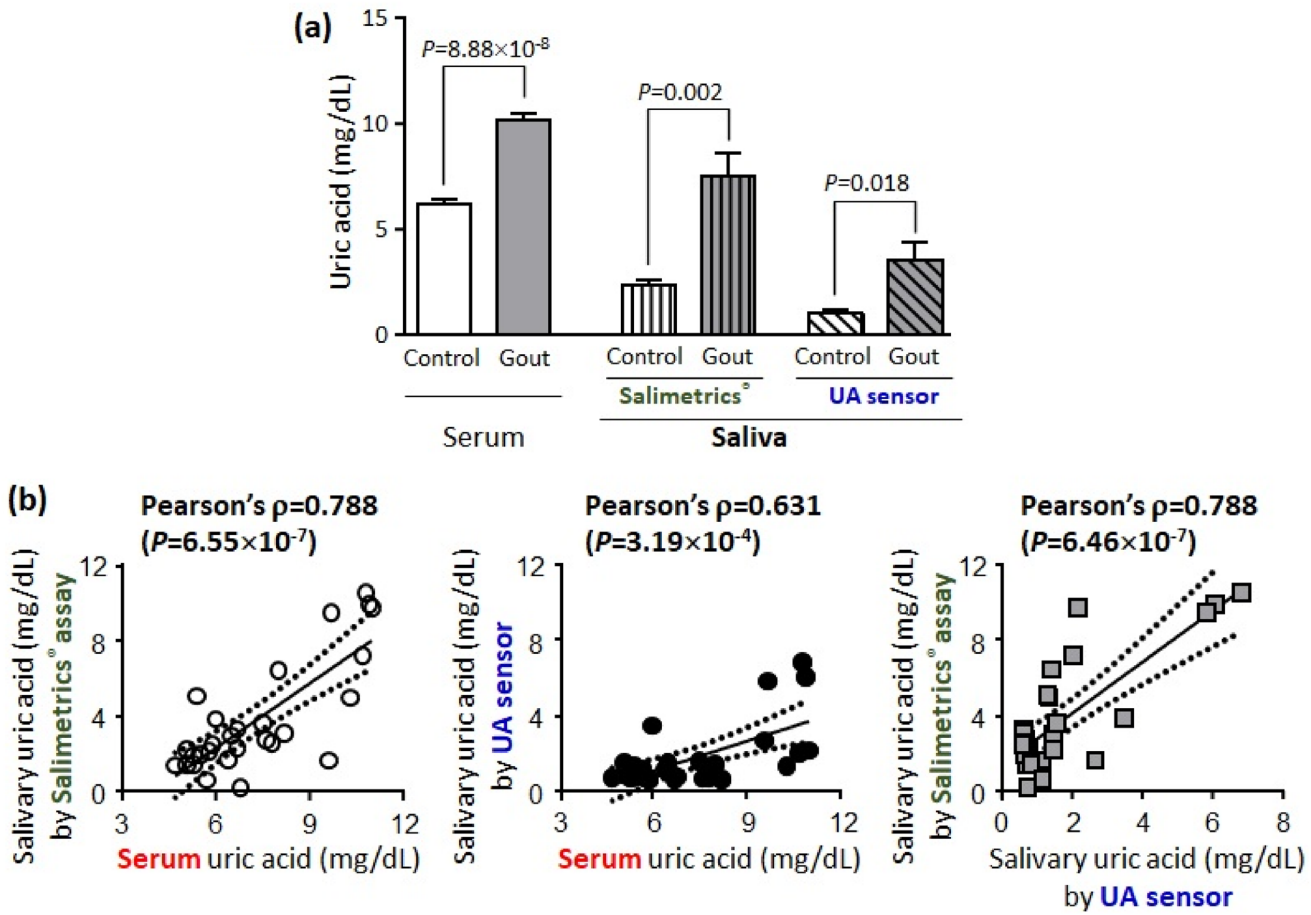
Uric acid (UA) levels in serum and unstimulated whole saliva samples. (**a**) Patients with gout (n = 8) showed significantly higher levels of serum or salivary UA than control subjects (n = 20, all *p* < 0.05). Salivary UA levels measured with our UA sensor were significantly lower than serum and salivary UA levels measured via conventional enzymatic colorimetric assays in both controls and gout cases (both *p* < 0.0001 by ANOVA). Error bars represent standard error of mean. (**b**) UA levels in serum or saliva (measured with Salimetrics® and UA sensor) were significantly positively correlated with each other.

First, serum UA levels were significantly higher in patients with gout (10.23 ± 0.36 mg/dL, mean ± SEM) than in non-gout controls (6.20 ± 0.22 mg/dL, *p* = 8.77 × 10^−10^; Fig. 4a). Hyperuricemia (defined as serum UA ≥ 7.0 mg/dL) was significantly more prevalent in the gout group than in the control group (100% vs. 20%, *p* = 1.59 × 10^−10^). Urate-lowering therapy decreased serum UA in 3/8 (37.5%) gout patients to concentrations lower than 6.0 mg/dL.

Next, salivary UA levels were 2.34 ± 0.25 mg/dL in the control group and 7.51 ± 1.09 mg/dL in the gout group based on Salimetrics® assay (*p* = 0.002, Fig. 4a); UA levels in saliva were 37.7% lower in the sera of controls and 73.4% in the sera of gout patients. In case of the fabricated UA sensor, the salivary UA levels were 1.05 ± 0.15 mg/dL in the control group and 3.54 ± 0.81 mg/dL in the gout group (*p* = 0.018; Fig. 4a). These salivary levels corresponded to 34.6% and 16.9% of serum UA levels in the gout and control groups, respectively. In both methods, salivary UA levels were significantly higher in the gout group than in the control group, although they were significantly lower than serum UA levels. The results were consistent with those of previous studies^11–14^. However, salivary UA levels based on Salimetrics® assay were significantly higher than those obtained with the prepared UA sensor involving all the subjects (*p* = 5.26 × 10^−13^), particularly high in the low concentration range below 2 mg/dL by UA sensor (Fig. 4b). This difference of salivary UA levels between two methods may be attributed to the large variability of the enzymatic test kit to low concentration of UA, together with interferences due to endogenous compounds. And it may also come from the reduced diffusion of signal molecules in the prepared UA sensor. First, the Salimetrics® assay utilizes an enzymatic reaction mixture that enables the detection of UA by the production of a red chromogen. UOx is oxidize UA to allantoin and H_2_O_2_. And then H_2_O_2_ is used in the second enzymatic reaction by peroxidase to form a chromogen which is quantitatively measured at a wavelength of 515 (or 520) nm. Although this enzymatic assay is simple and commercially available, the chromogen formation by peroxidase-catalyzed reaction of H_2_O_2_ is susceptible to interference from endogenous confounding components in biological matrixes, and eventually may cause false positive or negative results^19–22^. The Salimetrics® assay requires centrifugation to remove mucins and other interferents from saliva samples, but it cannot perfectly eliminate multiple compounds that can act as interferers. Second, we evaluated inter-day precision using UA standard (5 mg/dL) and controls (High & Low) in the same kit according to the manufacturer’s instructions. As shown in Table S2, the CV for UA standard which was used for the calculation of UA concentration was 8.8% and control-High (estimated to be 8.6 mg/dL) showed less than 5% CV. But the CV for control-Low (estimated to be 2.5 mg/dL) was as much as 36% (Table S2). This result may be attributed to the relatively low sensitivity to low concentrations of UA. And it may cause some limitations of enzymatic assays such as the large deviation of test results and kit-to-kit variability, which have been mentioned in several studies^2,19,24,50^. On contrary, the CV of UA sensor was all less than 5% at low concentration range from 1.7 to 5 mg/dL (Table S3). And the prepared UA sensor, UOx-paper/PPD/PrB-SPCE which did not use peroxidase, was fabricated with two types of membrane including UOx paper and PPD to minimize the interference due to multiple components within saliva. But these protecting layers may reduce the diffusion of the signal molecule, resulting in decreased UA levels. However, the salivary UA levels measured in the control group are in agreement with previous studies analyzed by HPLC-UV or electrochemical method^14,29,50^. Additionally, the mean salivary UA level in the gout group using the UA sensor was 3.37 times higher than in the control group, which was similar to 3.21 times in Salimetrics® assay. The UA levels in serum and saliva measured via two methods were significantly correlated with each other (Pearson correlation coefficients ranged from 0.631 to 0.788, Fig. 4b).

**Figure 5:**
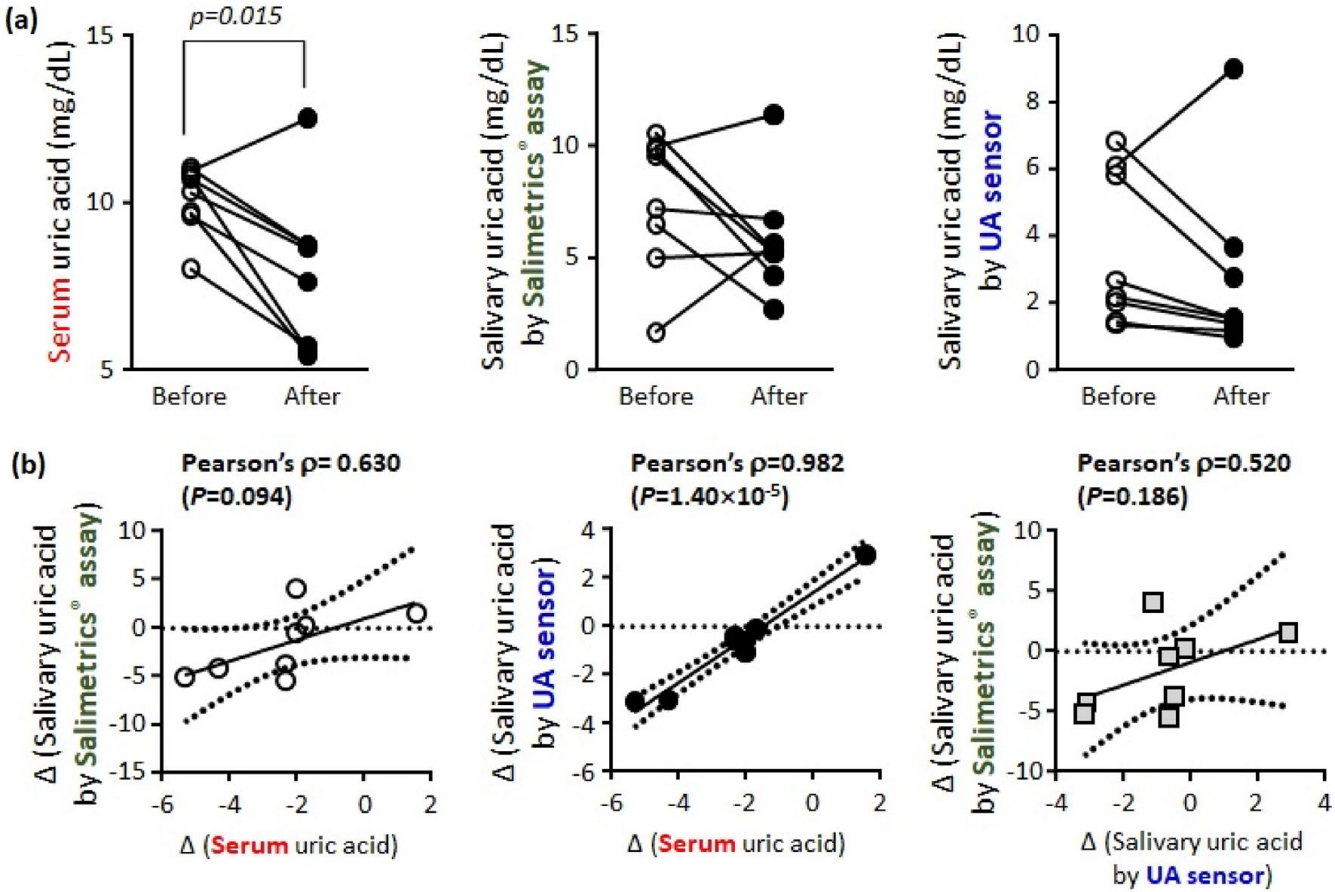
Change in uric acid (UA) levels in serum and unstimulated whole saliva samples after urate-lowering therapy. (**a**) In patients with gout (n = 8), serum UA levels decreased significantly after the treatment. However, salivary UA levels were not significantly changed regardless of the method used. (**b**) Changes in serum UA were significantly correlated with changes in salivary UA levels measured with our UA sensor (*p* < 0.0001), but not Salimetrics® assay.

Urate lowering therapy significantly reduced serum UA levels in the gout group from 10.23 ± 0.36 mg/dL to 7.84 ± 0.84 mg/dL (*p* = 0.015) as shown in Fig. 5a. Although salivary UA levels tended to decrease from 7.51 ± 1.09 to 5.78 ± 0.90 mg/dL in the Salimetrics® assay and from 3.54 ± 0.81 to 2.75 ± 0.95 mg/dL with the UA sensor, no statistical significance was observed. As shown in Fig. 5b, when using Salimetrics® assay, the changes in salivary uric acid level were not correlated with that of serum UA levels. Nevertheless, our UA sensor revealed that change in salivary UA levels was significantly correlated with change in serum UA levels (Pearson correlation coefficient = 0.982). It might be attributed to reduced interference of UA sensor, resulting in accurate detection of UA in saliva, even at the low concentration range of UA. As a result, the proposed UA sensor is highly reliable for the measurement of salivary UA. The advantages of our UA sensor, UOx-paper/PPD/PrB-SPCE, include: (1) sensitivity and specificity for UA in undiluted human saliva; (2) detection of UA in small volume (20 μL) of saliva sample; (3) rapid detection (within only 2 min) of UA (incubation 1 min + detection 1 min), and the ease of use. Therefore, the fabricated UA sensor can be utilized as a simple, fast, and reliable tool to determine salivary UA levels, which can reflect the serum UA level.

## Conclusions

In summary, we introduced a simple and reliable electrochemical UA sensor using UOx-paper integrated with PPD/PrB-SPCE. The UOx-paper acts as the sample absorption area for reaction between UOx and UA, as well as serving as a filter to prevent interference within saliva. PPD improves the selectivity and anti-biofouling of the electrode. As a result, the prepared UOx-paper/PPD/PrB-SPCE showed robust sensitivity for UA, with a wide linear range, high selectivity, and good reproducibility, and requires low sample volume. Using this UA sensor, we measured UA levels in undiluted, unstimulated saliva, subsequently comparing serum UA levels with those based on conventional UA assays (Salimetrics®). Although salivary UA levels determined by Salimetrics® assays were significantly higher than those measured with our UA sensor, they were significantly correlated with serum UA levels. Additionally, our UA sensor showed that changes in salivary UA level reflect changes in serum UA levels more closely than the Salimetrics® assay. Therefore, we expect that the prepared UA sensor, UOx-paper/PPD/PrB-SPCE, will be utilized in POCT to evaluate salivary UA levels in subjects with UA-associated diseases.

## Supplementary Information

Supplementary Information.

## Data Availability

The datasets used and/or analyzed during the current study are available from the corresponding author upon reasonable request.
