# Supplementary material for: Electrochemical detection of uric acid in undiluted human saliva using uricase paper integrated electrodes
Source: Sci Rep. 2022 Jul 14;12:12033. doi: 10.1038/s41598-022-16176-5 (PMC9283454; doi:10.1038/s41598-022-16176-5)
Supplement: Supplementary file 1 — Supplementary Information. [file 41598_2022_16176_MOESM1_ESM.pdf]

Supplementary Information

Electrochemical detection of uric acid in undiluted human saliva using uricase paper integrated electrodes

Seong Hyun Han[1], You-Jung Ha[2], Eun Ha Kang[2], Kichul Shin[3], Yun Jong Lee[2,4,*], Gi-Ja Lee[1,5,*]

[1] Department of Medical Engineering, Kyung Hee University, Graduate School, Seoul 02447, Korea

[2] Division of Rheumatology, Department of Internal Medicine, Seoul National University Bundang Hospital, Seongnam-si, Gyeonggi-do 13620, Korea

[3] Division of Rheumatology, Seoul Metropolitan Government-Seoul National University Boramae Medical Centre, Seoul 07061, Korea

[4] Department of Medical Device Development, Seoul National University Graduate School, Seongnam-si, Gyeonggi-do 13605, Korea

[5] Department of Biomedical Engineering, College of Medicine, Kyung Hee University, Seoul 02447, Korea

* To whom correspondence should be addressed. E-mail: yn35@snu.ac.kr & gjlee@khu.ac.kr

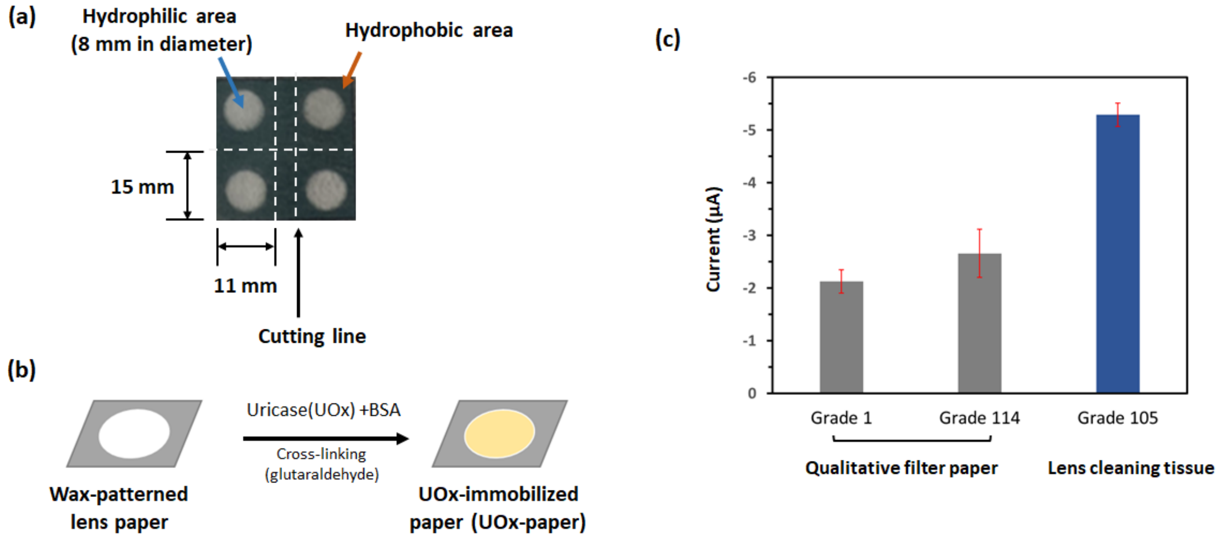

Figure S1. (a) A photographic image of wax-patterned paper substrate. (b) Schematic illustration of the fabrication process of the UOx-paper. (c) The current response of UOx-paper/PPD/PrB-SPCE to 1000 μM uric acid at -0.1 V (vs. Ag pseudo-reference electrode) according to the type of Whatman® paper.

Table S1. Characteristics of Whatman® paper

Type	No.	Pore size (μm)	Thickness (μm)	Characteristics
Filter paper	Grade 1	11	180	The most widely used filter paper for routine applications with medium retention and flow rate
	Grade 114	25	190	High wet strength due to the addition of a small quantity of chemically stable resin
Lens cleaning tissue	Grade 105	-	35~40	Chemically pure and free from silicones or other additives/very strong without residual fibers

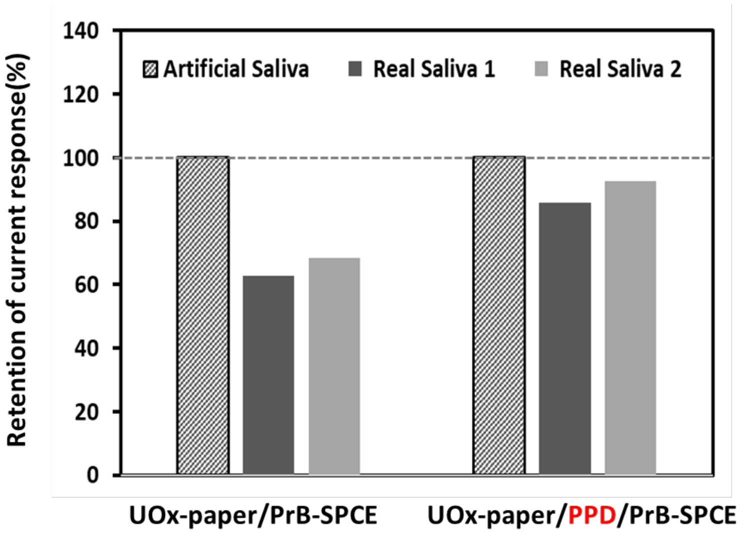

Figure S2. Retention (%) of current response to uric acid (UA, 300 μM) of UOx-paper/PrB-SPCE (without PPD) and UOx-paper/PPD/PrB-SPCE (with PPD) in artificial saliva and real saliva samples. It was presented as the relative percentage of current by UA in real saliva to that in artificial saliva.

Table S2. Inter-day precision of Salimetrics® uric acid enzymatic assay kit. The average blanked optical density (OD) was represented as a subtraction of the average OD for blank wells from the OD of the standard and controls (High & Low), respectively.

Trial No.	Blank			Standard (5 mg/dL)				Control-Low				Control-High			
	Optical density (OD) at 515 nm														
	**B-1**	**B-2**	**Average OD**	**STD-1**	**STD-2**	**Average OD**	**Average blanked OD**	**Con L-1**	**Con L-2**	**Average OD**	**Average blanked OD**	**Con H-1**	**Con H-2**	**Average OD**	**Average blanked OD**
1	0.079	0.079	0.079	0.202	0.205	0.204	0.1245	0.099	0.098	0.099	0.0195	0.357	0.355	0.356	0.3365
2	0.078	0.081	0.080	0.195	0.200	0.198	0.1180	0.101	0.105	0.103	0.0235	0.314	0.354	0.334	0.3105
3	0.083	0.075	0.079	0.180	0.191	0.186	0.1065	0.099	0.102	0.101	0.0215	0.329	0.335	0.332	0.3105
4	0.075	0.094	0.085	0.208	0.208	0.208	0.1235	0.102	0.088	0.095	0.0105	0.342	0.356	0.349	0.3385
5	0.075	0.073	0.074	0.209	0.211	0.210	0.1360	0.101	0.111	0.106	0.0320	0.358	0.352	0.355	0.3230
						**Mean**	0.122			**Mean**	0.021			**Mean**	0.324
						**STDEV***	0.011			**STDEV**	0.008			**STDEV**	0.014
						**%CV#**	**8.815**			**%CV**	**36.136**			**%CV**	**4.177**

*STDEV: standard deviation; #CV: coefficient of variation

Table S3. Inter-day precision of UOx-paper/PPD/PrB-SPCE by measuring the current response to UA (100, 200, and 300 μM) using 5 sensors on different fabrication dates.

Trial No.	Current response to UA (μA)		
	100 μM UA (1.7 mg/dL)	200 μM UA (3.3 mg/dL)	300 μM UA (5 mg/dL)
1	-0.814	-1.341	-1.916
2	-0.870	-1.293	-1.721
3	-0.893	-1.387	-1.862
4	-0.816	-1.323	-1.808
5	-0.896	-1.347	-1.760
**Mean**	-0.858	-1.338	-1.813
**STDEV***	0.040	0.034	0.078
**%CV#**	4.704	2.573	4.299

*STDEV: standard deviation; #CV: coefficient of variation
